# Cognitive Effort during Visuospatial Problem Solving in Physical Real World, on Computer Screen, and in Virtual Reality

**DOI:** 10.3390/s24030977

**Published:** 2024-02-02

**Authors:** Raimundo da Silva Soares, Kevin L. Ramirez-Chavez, Altona Tufanoglu, Candida Barreto, João Ricardo Sato, Hasan Ayaz

**Affiliations:** 1 School of Biomedical Engineering, Science and Health Systems, Drexel University, Philadelphia, PA 19104, USA; klr355@drexel.edu (K.L.R.-C.); at3469@dragons.drexel.edu (A.T.); cd3272@drexel.edu (C.B.); 2 Center of Mathematics Computation and Cognition, Universidade Federal do ABC, São Bernardo do Campo 09606-405, Brazil; joao.sato@ufabc.edu.br; 3 Department of Psychological and Brain Sciences, College of Arts and Sciences, Drexel University, Philadelphia, PA 19104, USA; 4 Drexel Solutions Institute, Drexel University, Philadelphia, PA 19104, USA; 5 A.J. Drexel Autism Institute, Drexel University, Philadelphia, PA 19104, USA; 6 Department of Family and Community Health, University of Pennsylvania, Philadelphia, PA 19104, USA; 7 Center for Injury Research and Prevention, Children’s Hospital of Philadelphia, Philadelphia, PA 19104, USA

**Keywords:** geometry puzzle, tangram, spatial cognition, cognitive workload, virtual reality, fNIRS, neuroergonomics

## Abstract

Spatial cognition plays a crucial role in academic achievement, particularly in science, technology, engineering, and mathematics (STEM) domains. Immersive virtual environments (VRs) have the growing potential to reduce cognitive load and improve spatial reasoning. However, traditional methods struggle to assess the mental effort required for visuospatial processes due to the difficulty in verbalizing actions and other limitations in self-reported evaluations. In this neuroergonomics study, we aimed to capture the neural activity associated with cognitive workload during visuospatial tasks and evaluate the impact of the visualization medium on visuospatial task performance. We utilized functional near-infrared spectroscopy (fNIRS) wearable neuroimaging to assess cognitive effort during spatial-reasoning-based problem-solving and compared a VR, a computer screen, and a physical real-world task presentation. Our results reveal a higher neural efficiency in the prefrontal cortex (PFC) during 3D geometry puzzles in VR settings compared to the settings in the physical world and on the computer screen. VR appears to reduce the visuospatial task load by facilitating spatial visualization and providing visual cues. This makes it a valuable tool for spatial cognition training, especially for beginners. Additionally, our multimodal approach allows for progressively increasing task complexity, maintaining a challenge throughout training. This study underscores the potential of VR in developing spatial skills and highlights the value of comparing brain data and human interaction across different training settings.

## 1. Introduction

Spatial cognition has been found to be strongly associated with academic achievement in STEM (science, technology, engineering, and mathematics) fields [1,2,3]. The connection between spatial skills and math ability has been one of the most robust findings in cognitive psychology [4]. Educational research contributions to understanding spatial cognition have become increasingly important as educators and policymakers seek to develop evidence-based methods and curricular materials to enhance teaching–learning [2,5]. Therefore, thinking about technological solutions to help learn challenging domains, such as mathematical thinking or geometry concepts, is even more essential.

Visuospatial training is practical, durable, and transferable [6]. A comprehensive meta-analysis of 217 studies revealed a significant finding: spatial skills can be effectively enhanced across all age groups through targeted training interventions [6]. Similarly, empirical data indicate that enhancing visuospatial skills can significantly improve general mathematical aptitude and specific domains such as geometry [7]. The evidence strongly supports implementing formal programs to nurture and refine spatial abilities. However, it is important to note that not all spatial cognition training is effective [8]. Therefore, it is crucial to have a solid understanding of visuospatial protocols to ensure that students can benefit from the practice. Investigating visuospatial processes, such as translation, symmetry, and area, through conventional means (thinking aloud, post-tests) presents a significant challenge in academic research since subjects often have difficulty in producing verbal accounts for their actions [9]. In this sense, neuroscience studies have employed neuroimaging techniques to investigate mental effort signs and elucidate the neural mechanisms that underlie cognitive effort during spatial reasoning.

Functional magnetic resonance imaging (fMRI) is one of the established methods of neuroimaging that can be valuable for understanding brain activity mapping development [10,11,12] and for the investigation of visuospatial cognition [13,14]. For example, an fMRI study demonstrated that subjects who underwent a three-month intervention with Tetris game training showed decreased activation in the frontal cortex area [14]. Indeed, neuroimaging studies have consistently shown that some brain regions, including the superior parietal and the frontal cortices, were involved with many bilateral activation regions during mathematical [15,16,17] and visuospatial tasks [18,19].

Neuroimaging studies applying fMRI have significantly impacted our understanding of brain function. However, this technique has limitations, such as high operation costs, limiting the participants’ movement, and the fact that it is very susceptible to motion artifacts. All these together make fMRI challenging to use in more naturalistic experiments outside of traditional labs, especially when working with children [20,21]. Alternatively, one optical neuroimaging technique is known as functional near-infrared spectroscopy (fNIRS) [22,23,24,25]. It measures a similar cortical hemodynamic signal as fMRI but with a wearable and potentially mobile form factor, and it has the advantages of being a low-cost, safe, minimally intrusive, and non-invasive approach [26]. This neuroimaging technique has been applied in increasingly naturalistic paradigms [27,28] consistent with neuroergonomics, the study of the brain in everyday life [29]. In particular, it has been utilized in educational research, such as learning [25,27,28,30], problem-solving [31,32], and authentic educational paradigms [24,33,34,35]. Furthermore, fNIRS has also been applied in other naturalistic neuroscience studies with complex real-world tasks [21,36,37,38,39,40,41] and is a valuable technological tool for investigating cognitive aspects in realistic educational interactions [35,42].

A growing number of fNIRS studies have shown that the modulation of activity in the frontal cortex reflects the cognitive effort under cognitive demands in some conditions [28,31,43,44,45,46,47]. For example, a study demonstrated that the prefrontal activation intensity measured by fNIRS sheds insight into the level of mental effort, indicating engagement with demanding tasks in a flight simulator [47]. Another study showed through fNIRS that good problem-solvers increasingly had lower oxygenation levels in the prefrontal cortex (PFC) than average problem-solvers during tangram puzzles [32]. The findings suggest less of a dependence on the neural resources in the cortical regions recruited during the subsequent visuospatial puzzles. Taken together, the decrease in cortical activation indicates changes in neural networks due to the development of visuospatial ability and learning throughout the training protocol. Furthermore, using fNIRS technology has also improved the assessment of cognitive effort by calculating neural efficiency [48,49]. Such a method combines measurements of behavioral output and PFC activity to determine mental effort in relation to performance [50,51]. In fact, neural efficiency provides valuable insights into the brain’s activity during various cognitive tasks and has led to a deeper understanding of cognitive processes [51], which can help in evaluating learning materials and instructional designs.

In addition to neuroimaging techniques, virtual reality (VR) is also considered a great promising tool to improve research on spatial cognition. It brings realistic scenarios with controlled stereoscopic displays that can enhance the understanding and learning of 3D objects/environments [52]. It has been suggested that immersive virtual environment training leads to a lower cognitive load and more efficient visual stimuli processing for users than two-dimensional content [53]. For example, a study revealed that participants performed spatial reasoning tasks, specifically paper-folding tasks, with greater accuracy and speed in VR [53]. In addition, another study showed that participants performing visuospatial reasoning tasks (e.g., mental rotation task, MRT) in immersive VR (which displayed a 3D visualization) reached a better score and average time per question than performing the same task with 2D stimuli (screen display), suggesting that the processing and comprehension of 3D stimuli in VR environments are more effective than perceiving 2D stimuli of 3D models [54]. Despite some positive results, VR in the learning context is still being discussed, as virtual training could also result in high workloads for students, leading to negative learning experiences [55]. Therefore, adapting the VR environment is necessary for the user’s well-being and productivity. In this context, neuroscience could provide helpful information on brain activation and visuospatial tasks. Together, VR technology and neuroimaging techniques are valuable for assessing mental effort signs to evaluate visuospatial reasoning tasks in distinct scenarios.

In this study, a comparison of a task presentation in VR, on a computer screen (CS), and in physical real-world (RW) mediums was investigated to (1) compare the brain activity related to the cognitive workload and neural efficiency during easy and difficult visuospatial tasks with and without VR settings and to (2) evaluate if VR-based visualizations have any influence on enhancing visuospatial task performance. Here, we aimed to assess cognitive effort by analyzing the brain’s hemodynamic activity in participants while they engaged in solving geometry puzzles under three distinct conditions: virtual reality, computer screen, and real-world settings.

## 2. Materials and Methods

### 2.1. Participants

Thirty healthy participants (fifteen females and fifteen males) included in these analyses had a mean (SD) age of 24.3 (5.30) years. Each participant attended one session and completed all three phases: VR settings, RW settings with solid puzzle parts, and CS, a typical two-dimensional computer screen setup, at Drexel University’s Neuroergonomics and Neuroengineering for Brain Health and Performance Research Lab at the CoNQuER Collaborative. All participants provided written informed consent that was approved by Drexel University’s Institutional Review Board and received monetary compensation for their time. All participants involved in the study were found to meet the eligibility criteria for right-handedness as per the Edinburgh Handedness Inventory. Additionally, they exhibited correctable 20/20 vision, no history of brain injury or psychological disorder, and were not under the influence of medication that could potentially impact brain function. We adopted similar selection criteria to a previous study involving biomarker measurements with fNIRS during virtual reality tasks [56]. We selected 18 as the minimum age to target adults. We selected a maximum of 40 years old to eliminate primarily generational differences and potential age-related confounding factors in cognitive function. The invitation for participants was sent to the Drexel University community, and we were careful to maintain balance in our sample regarding characteristics such as age, gender, video game experience, and handiness. To ensure a balanced sample, we utilized a background questionnaire.

### 2.2. VR Equipment

The hardware equipment used in the VR design was an Oculus Rift headset (Oculus Rift VR, Meta Platforms Inc., Menlo Park, CA, USA) with a sensor to track the user’s hand movements during the task. The VR equipment was connected to a laptop computer. The software used for the immersive VR application was Cubism VR in its 1.6.1 version. It presented the puzzles during the VR and the CS phases, with all task events recorded, including the start and end of each puzzle and the participants’ movements, allowing for behavioral analysis and brain synchronization.

### 2.3. Behavioral Measurements

The behavioral responses were recorded using a video camera (Logitech HD C270, Logitech Inc., Fremont, CA, USA) to evaluate the problem-solving process across the mediums. The number of actions was defined by how many times the participant placed the piece to solve the puzzle. The number of rotations was how often the participant rotated the pieces during the problem-solving. The number of mistakes means how many times the participant placed the piece in the wrong position to solve the puzzle, and the number of give-ups indicates how many times the participant tried to place the piece but gave up by stopping holding the piece to try another approach to solve the puzzle.

### 2.4. fNIRS Data Acquisition

**Figure 1:**
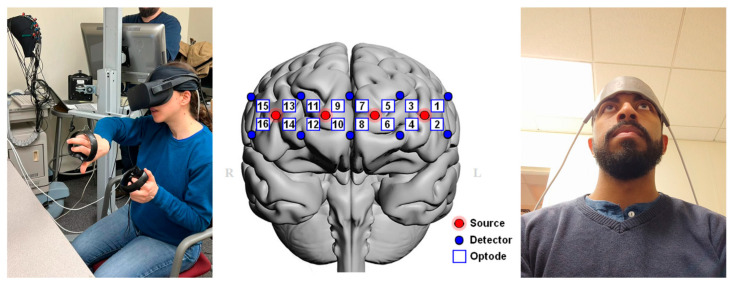
Wearable fNIRS sensor for the monitoring of brain activity during problem-solving under the VR headset (**left**). Layout of the fNIRS sensor pad with 16 optodes in the anterior prefrontal cortex (**middle**) and position over the forehead region with anatomical landmarks (**right**). The red dots represent the light sources, while the blue dots indicate the light detectors used in fNIRS measurements, and the measurement region was in between each light source and detector pair.

We employed a wearable flat fNIRS sensor pad (Imager Model 1100 by fNIR Devices, LLC—Potomac, MD, USA) that used near-infrared light to monitor the hemodynamics (oxygenated and deoxygenated hemoglobin) in the prefrontal cortex [27]. The LED illumination sources emitted two wavelengths of near-infrared light (730 nm and 850 nm), and the signals were recorded by the COBI Studio software (v1.5.0.55) at a sampling rate of 2 Hz with 10 photodetectors, resulting in 16 optodes (cortical regions, see Figure 1) being monitored as described by [27]. The sensor locations for the cortical signals were obtained from areas previously reported in the literature as brain regions related to cognitive effort during visuospatial tasks [31,32].

### 2.5. Visuospatial Task

**Figure 2:**
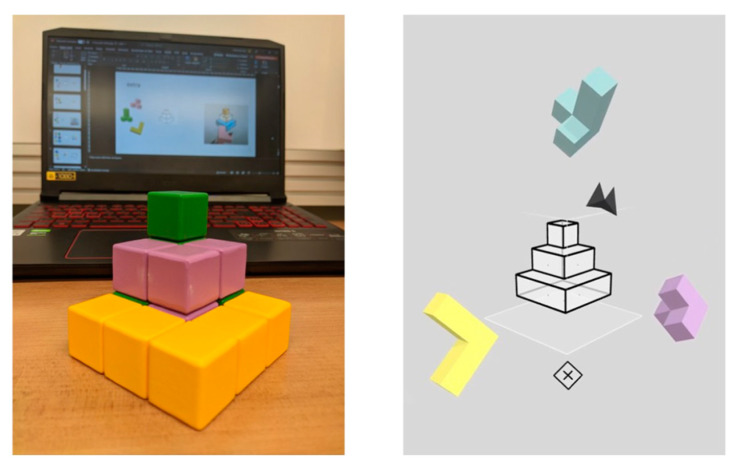
Geometric puzzles presented in the real world, in virtual reality, and on computer screen.

We used tangram geometric puzzles based on protocols designed at Drexel University that require visuospatial reasoning [30,31,32]. The three-dimensional puzzles were built into a virtual editor (Cubism VR) that could be implemented within physical geometric puzzle pieces constructed from solid wood building blocks (see Figure 2). Regarding the difficulty level, it increased with the number of components, required movements, rotations, and complexity of the target shape. Therefore, easy puzzles were flat with fewer pieces and spread out with individual piece silhouettes that could be identified as a jigsaw, whereas more challenging puzzles included pyramids, squares, and other compact shapes with more folds and more pieces without a clue of how the pieces needed to be placed in the larger area.

Regarding the geometric puzzle, we used 3D puzzles built into a virtual editor that could be performed in solid wood pieces. The puzzle task required the participants to assemble the blocks into predefined geometric shapes, testing the participants’ spatial thinking skills. The participants picked up the puzzle pieces and put them together to form a predefined shape, which appeared as a transparent template. The puzzle pieces were floated in front of the participants so they could pick them up with the controllers, view them from all sides, and freely place them in a template space to fit the blocks until every space was covered. At the difficult level, it increased the number of components, folds, the size, and the complexity of the target shape. During each puzzle, the participants were presented with pieces of the geometry puzzle and asked to build a geometric form. Then, the participants solved the geometry challenges.

**Figure 3:**
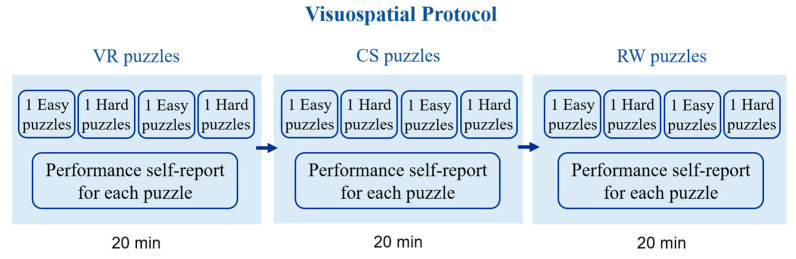
The visuospatial protocol was a three-phase session that utilized virtual reality, computer screens, and real-world scenarios in counterbalanced order across participants to solve geometry puzzles.

By ending each puzzle, the participants were asked about the effort required during the task. After finishing the first phase of the session, the participants solved the same kind of geometry puzzles but in a different medium (VR, CS, or RW environment) in the second and third phases of the session (Figure 3).

### 2.6. Experimental Setup

The task was performed in the Neuroergonomics and Neuroengineering for Brain Health and Performance Research Laboratory of the School of Biomedical Engineering, Science and Health Systems at Drexel University. Each subject participated in the task individually in a session lasting approximately 60 min. We used a camera to record the computer screen and the solid puzzles on the table. The participants were aware that they were being recorded. We removed any identifiable information from the video or transcriptions.

After receiving the information, the participants were allowed to practice the task before starting the actual study session. The practice required all the shape manipulation without problem-solving since each piece’s target location was identified. An fNIRS headband sensor pad was placed on the participants’ foreheads to continuously track brain hemodynamics related to cognitive function. The participants were also fitted with a VR headset for the virtual reality task over the sensor. Before the session, we asked the participants about their background information, such as their video game experience, age, and gender. Then, we explained the geometry puzzle task and procedure. During the experiment, we used a camera directed at the puzzles (presented by a computer screen or solid puzzle on the table) to record the problem-solving process and analyze each participant’s behavior.

After setup, the participants were instructed and familiarized themselves with the puzzles. The participants were asked to conduct several shape manipulations without needing problem-solving to accustom themselves to the experiment settings. Once the participants felt comfortable, we started the experimental session (VR, RW, or CS task) and monitored the brain with an fNIRS device. We kept the task order balanced. The participants were given geometry pieces during each puzzle and asked to build a specific geometric form (Figure 1).

The participants were given three minutes to solve one “easy” puzzle and six more minutes to solve one “difficult” puzzle. By concluding each puzzle, the subjects were asked to answer a short self-report survey regarding their performance and mental effort. The survey consisted of three items using a 0–10 Likert scale to evaluate subjective cognitive effort after each puzzle (How difficult was the last game? How did you perform in the last game? How much mental effort did you apply to reach that performance in the last game?). The participants were given 30 s to respond to all the questions. The order of the puzzles was one easy puzzle followed by one difficult puzzle until completing four puzzles in each phase of the session. By ending the first part of the session, the participants started the second and third phases of the session following the same procedure but in a different setup (CS, RW, or VR environment) until they completed the three phases of the session. Each phase had two easy and two difficult puzzles, resulting in twelve challenges during the session.

### 2.7. Data Analysis

The raw light intensity signals were processed using a lowpass finite impulse response (FIR) filter with a cutoff of 0.1 Hz to attenuate the physiological artifacts and high-frequency, respiratory, and cardiac noises [57,58]. Then, we used the sliding-window motion artifact rejection (SMAR) algorithm to remove motion artifacts and potential saturations [59]. Next, the fNIRS data for each block were extracted using time synchronization markers for the start and end of the tasks during the experiment. Then, the modified Beer–Lambert law (MBLL) with a local baseline at the beginning of each task period was applied to convert the optical signals of each wavelength (760 and 850 nm) to changes in the concentration of oxygenated hemoglobin (HbO) and deoxygenated hemoglobin (HbR), as well as a summation of the two, resulting in total hemoglobin (HbT) and the difference in hemoglobin (Oxy) as biomarkers. All biomarker concentration changes for each of the 16 optodes during each condition block were calculated separately from the pre-processed light intensity data via the MBLL for each block. For each condition block, the hemodynamic response of each optode was averaged over time, and the final output of each optode was the average of the biomarker. The false detection rate (FDR) was applied to control for type I errors [60,61], and we rejected the null hypotheses for FDR q < 0.05 across the optodes.

The PFC activity differences, behavioral outcomes, and self-assessment survey were compared statistically using linear mixed-effects models with repeated measures. We measured PFC activity by averaging the HbO levels. The performance was evaluated based on accuracy and the time that the participants spent solving the puzzle. For behavioral measurements, we considered the number of actions, rotations, mistakes, and give-ups. The model fixed terms were Medium + Difficulty + Medium*Difficulty, with a random subject term. Medium refers to the task presentation environment, including the virtual reality (VR), computer screen (CS), and real-world (RW) tasks. Difficulty levels included easy and hard. Post hoc comparisons were performed for all pairs of factor levels, and multiple comparisons were corrected with the Bonferroni method. All figures used the standard error of the mean (SEM).

A comprehensive metric was derived through the process of conducting the efficiency analysis to establish a correlation between cognitive effort and achievement results [62]. This study described the outcome by the changes in the time spent to solve the puzzles, while mental effort was assessed based on the changes in fNIRS-measured biomarkers. Effort (average of the biomarker; HbO) and behavioral (task performance score based on the time participants spent solving the puzzle) metrics were converted into Z-scored measurements, and then efficiency was computed using the distance of the point from the zero-efficiency line (i.e., where performance = effort) and assessed as dependent measurements in statistical tests [51]. Therefore, we used neural efficiency as a metric to compare neural activity performance.

## 3. Results

### 3.1. Self-Assessment Survey

**Figure 4:**
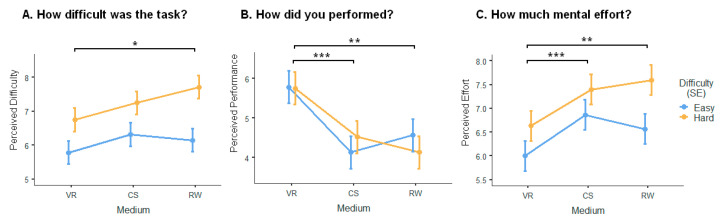
Self-report survey on subjective difficulty, performance, and mental effort in the difficult level of the puzzles and medium conditions. SEM, * *p* < 0.05, ** *p* < 0.01, and *** *p* < 0.001.

Within the survey, three distinct items (as shown in Figure 4) were employed to assess the participants’ perceptions of the difficulty level, performance, and mental effort required for each puzzle. These assessments were conducted utilizing a Likert scale, wherein a score of 0 indicated “extremely difficult”, “extremely poor”, and “extremely low”, while a score of 10 signified “extremely easy”, “extremely well”, and “extremely high”, respectively. The linear mixed-effect model revealed a significant effect on the participants’ perception of difficulty [F_(1, 325)_ = 27.661, *p* < 0.001] and medium [F_(2, 325)_ = 3.378, *p* < 0.05], indicating that the participants could differentiate between easy and difficult tasks across the different conditions. There was no significant interaction between medium and task difficulty. In addition, the perceived difficulty in VR significantly differed from that in the RW (Bonferroni’s post hoc test, t_(325)_ = −2.47, *p* < 0.05, Figure 4A). The perceived performance in VR significantly differed from that in the RW (Bonferroni’s post hoc test, t_(325)_ = 4.24, *p* < 0.001) and CS (Bonferroni’s post hoc test, t_(325)_ = 4.31, *p* < 0.001 in all cases, Figure 4B), and perceived effort in VR significantly differed from that in the CS (Bonferroni’s post hoc test, t_(325)_ = −3.73, *p* < 0.001, Figure 4C) and RW (Bonferroni’s post hoc test, t_(325)_ = −3.50, *p* < 0.01, Figure 4C). The results indicate that the participants believed that solving problems in the RW was more difficult than in VR. The survey also suggests that the performance perceived by the participants when solving the geometric puzzles in VR was better and required less mental effort compared to the other mediums. There was also a significant effect on the participants’ perception of their effort in terms of difficulty [F_(1, 325)_ = 16.861, *p* = 0.001] and medium [F_(2, 325)_ = 8.759, *p* < 0.001], indicating that the participants could differentiate the amount of effort required between easy and difficult tasks across the different mediums. There was no main effect of the interaction between medium and task difficulty on perceived effort. Finally, there was a difference in the perceived performance in terms of edium [F_(2, 325)_ = 12.2003, *p* < 0.001], but no main effect in terms of difficulty and their interaction.

### 3.2. Performance and Behavioral Measures

**Figure 5:**
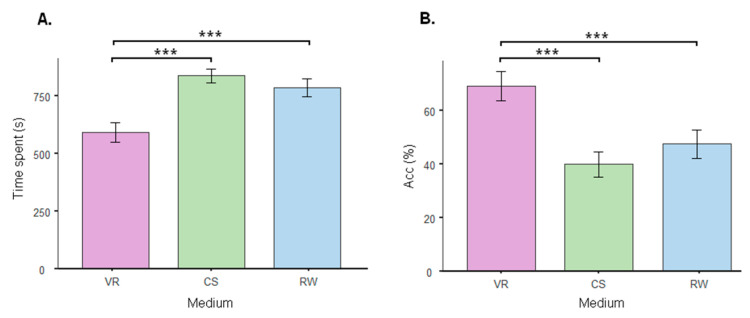
Performance in terms of time to solve the puzzles in seconds (**A**) and accuracy graph (**B**) showing the percentage of puzzles solved in virtual reality (VR), on computer screen (CS), and in real world (RW). The columns represent the mean, and the bars represent the standard error of the mean. *** *p* < 0.001.

The performance was evaluated based on the time spent solving the puzzle and accuracy. The linear mixed-effect model using medium (VR, CS, and RW) as fixed factors revealed a significant effect of medium on the time required to solve the puzzles [F_(2, 58)_ = 25.4, *p* < 0.001] and accuracy [F_(2, 58)_ = 15.2, *p* < 0.001]. In addition, the performance in VR significantly differed from that in the CS (time spent: Bonferroni’s post hoc test, t_(58)_ = −6.76, *p* < 0.001, Figure 5A; accuracy: Bonferroni’s post hoc test, t_(58)_ = 5.31, *p* < 0.001, Figure 5B) and RW (time spent: Bonferroni’s post hoc test, t_(58)_ = −5.31, *p* < 0.001, Figure 5A; accuracy: Bonferroni’s post hoc test, t_(58)_ = 3.95, *p* < 0.001, Figure 5B), indicating that the participants could solve more geometry tasks and solve such puzzles faster in VR than in the other mediums.

**Figure 6:**
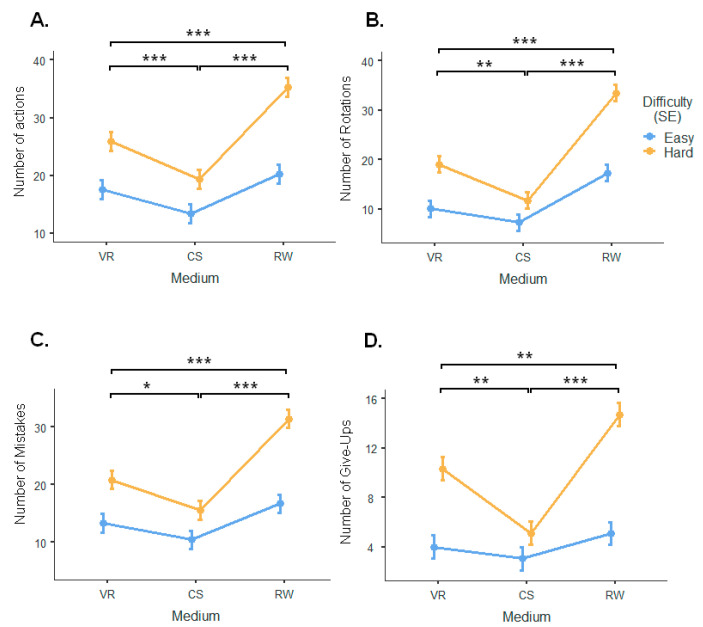
Behavior outcomes. Effect of difficulty and medium on the number of actions (**A**), number of rotations (**B**), number of mistakes (**C**), and number of give up (**D**). The bars represent the standard error. * *p* < 0.05, ** *p* < 0.01, and *** *p* < 0.001.

According to the linear mixed-effect model, there was a significant effect of difficulty on the number of actions [F_(1, 325)_ = 62.16, *p* < 0.001], number of rotations [F_(1, 354)_ = 52.38, *p* < 0.001], number of mistakes [F_(1, 325)_ = 60.50, *p* < 0.001], and number of give-ups [F_(1, 325)_ = 67.57, *p* < 0.001]. The linear mixed-effect model revealed a significant effect of medium on the number of actions [F_(2, 325)_ = 28.39, *p* < 0.001], number of rotations [F_(2, 354)_ = 47.18, *p* < 0.001], number of mistakes [F_(2, 325)_ = 30.45, *p* < 0.001], and number of give-ups [F_(2, 325)_ = 21.14, *p* < 0.001]. There was also a main interaction effect between medium and task difficulty on the number of actions [F_(2, 325)_ = 4.89, *p* < 0.01], number of rotations [F_(2, 354)_ = 6.18, *p* < 0.01], number of mistakes [F_(2, 325)_ = 6.16, *p* < 0.01], and number of give up [F_(2, 325)_ = 8.91, *p* < 0.001]. In addition, VR significantly differed from the CS (Bonferroni’s post hoc test, t_(325)_ = 3.53, *p* < 0.001 in the number of actions; t_(325)_ = 3.03, *p* < 0.01 in the number of rotations; t_(325)_ = 3.45, *p* < 0.01 in the number of give-ups; and t_(325)_ = 2.84, *p* < 0.05 in the number of mistakes, Figure 6A–D) and RW (Bonferroni’s post hoc test, t_(325)_ = −4.00, *p* < 0.001 in the number of actions; t_(325)_ = −6.48, *p* < 0.001 in the number of rotations; t_(325)_ = −3.05, *p* < 0.01 in the number of give-ups; t_(325)_ = −4.88, *p* < 0.001 in the number of mistakes, Figure 6A–D). Finally, the RW task significantly differed from the CS task (Bonferroni’s post hoc test, t_(325)_ = −7.53, *p* < 0.001 in the number of actions; t_(325)_ = −9.51, *p* < 0.001 in number of rotations; t_(325)_ = −6.50, *p* < 0.001 in the number of give-ups; and t_(325)_ = −7.71, *p* < 0.001 in number of mistakes, Figure 6A–D).

### 3.3. fNIRS Measures

**Figure 7:**
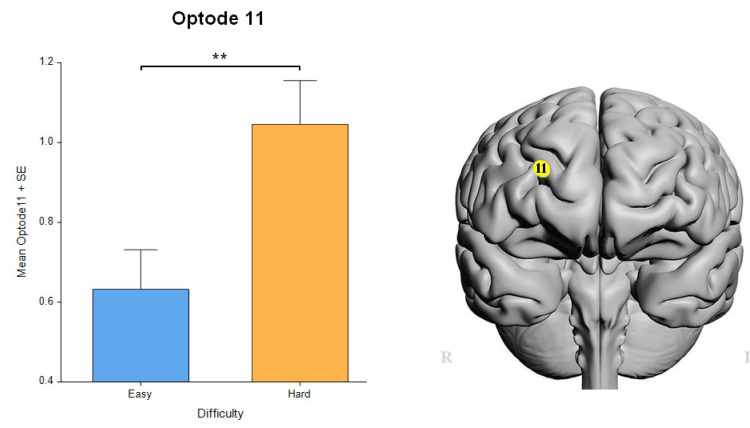
Neural measures on difficulty level, as expected, indicated higher activity during hard tasks. Oxygenated hemoglobin changes in optode 11 for easy tasks versus difficult tasks. Error bars: SEM, ** *p* < 0.01.

**Figure 8:**
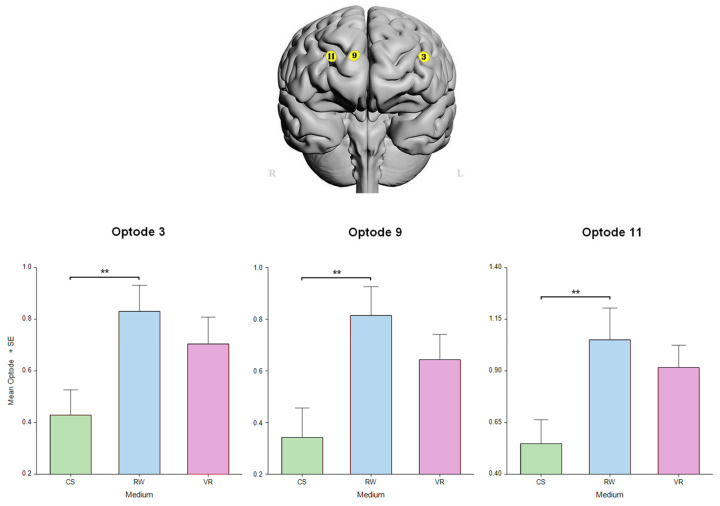
Neural measures on medium indicated the lowest activity during VR. Oxygenated hemoglobin changes in optodes 3, 9, and 11 for CS, RW, and VR mediums. Error bars: SEM, ** *p* < 0.01.

A significant difference in the main factor of difficulty was found only in the right medial PFC in optode 11 HbO [F_(1, 297.5)_ = 9.05, *p* < 0.01] that survived the FDR correction (Figure 7). The linear mixed-effect model revealed a significant effect of medium in the left dorsolateral PFC in optode 3 [F_(2, 310.2)_ = 4.3002, *p* < 0.05] and in the right medial prefrontal cortex in optode 9 [F_(2, 318.6)_ = 6.0004, *p* < 0.01] and optode 11 [F_(2, 299.1)_ = 4.791, *p* < 0.01], all FDR-corrected. In addition, these optodes, i.e., 3, 9, and 11, had post hoc significant differences between the CS and RW (*p* < 0.01, Figure 8).

### 3.4. Neural Efficiency Measures

**Figure 9:**
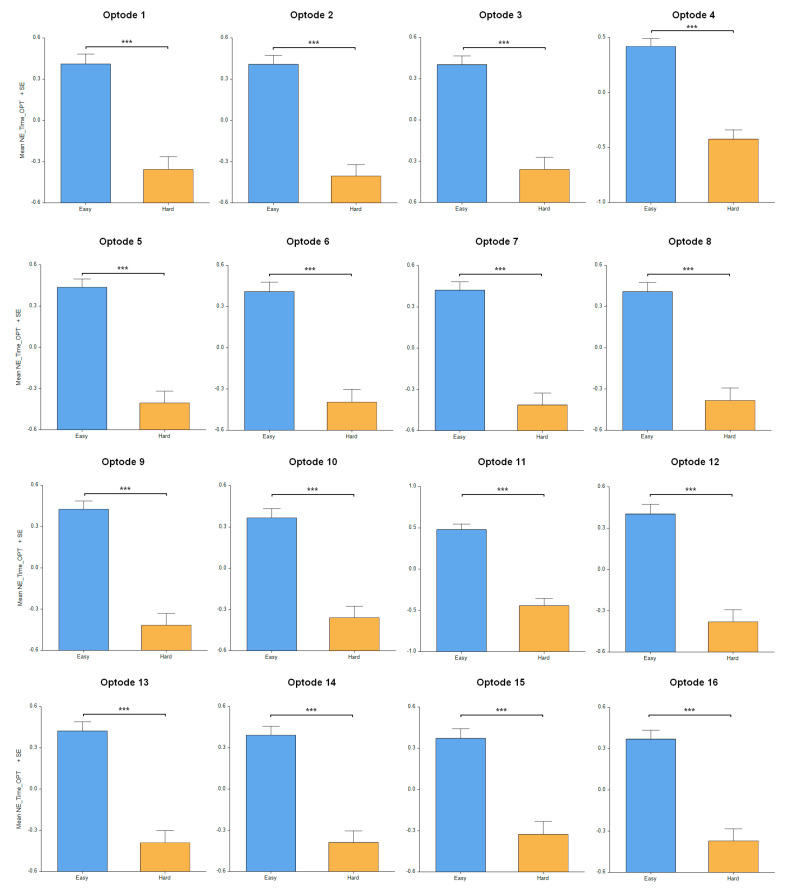
Neural efficiency measures on difficulty level with highest efficiency during easy tasks. Oxygenated hemoglobin changes in all 16 optodes for easy tasks versus difficult tasks. Error bars: SEM, *** *p* < 0.001.

The results in terms of neural efficiency (defined as combined neural activity performance, as shown in [51]) concerning the difficulty level of the puzzles had significant differences across all the PFC regions evaluated, including optode 1 [F_(1, 260.5)_ = 47.07, *p* < 0.001], optode 2 [F_(1, 319.4)_ = 65.61, *p* < 0.001], optode 3 [F_(1, 310.1)_ = 56.4, *p* < 0.001], optode 4 [F_(1, 291.6)_ = 65.09, *p* < 0.001], optode 5 [F_(1, 307.5)_ = 68.931, *p* < 0.001], optode 6 [F_(1, 274.7)_ = 52.78, *p* < 0.001], optode 7 [F_(1, 303.7)_ = 72.24, *p* < 0.001], optode 8 [F_(1, 278)_ = 53.63, *p* < 0.001], optode 9 [F_(1, 318.8)_ = 76.39, *p* < 0.001], optode 10 [F_(1, 309.6)_ = 49.82, *p* < 0.001], optode 11 [F_(1, 297.4)_ = 82.88, *p* < 0.001], optode 12 [F_(1, 301.1)_ = 52.68, *p* < 0.001], optode 13 [F_(1, 304.9)_ = 62.21, *p* < 0.001], optode 14 [F_(1, 312.5)_ = 61.46, *p* < 0.001], optode 15 [F_(1, 266.8)_ = 41.71, *p* < 0.001], and optode 16 [F_(1, 317.4)_ = 55.95, *p* < 0.001], as shown in the Figure 9. As expected, the participants solving the easy puzzles showed a higher neural efficiency in PFC activity compared to those solving the difficult geometric puzzles.

**Figure 10:**
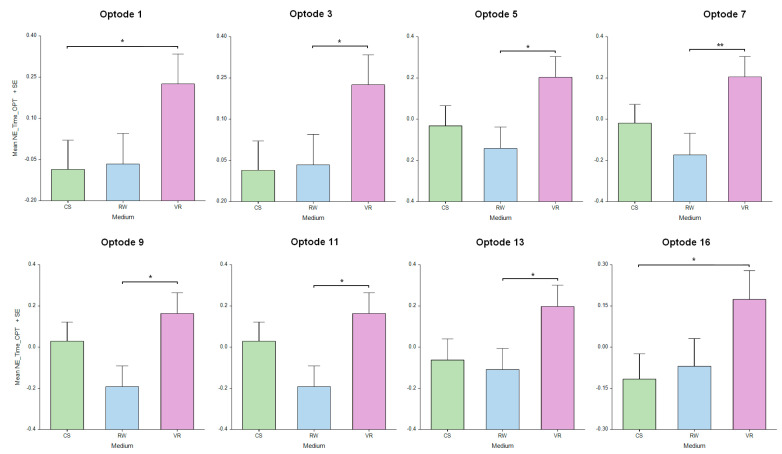
Neural efficiency measures on medium across the PFC brain region indicated the highest efficiency during VR. Oxygenated hemoglobin changes in optodes 1, 3, 5, 7, 9, 11, 13, and 16 for CS, RW, and VR mediums. Error bars: SEM, * *p* < 0.05, ** *p* < 0.01.

The results in terms of neural efficiency regarding *medium* had significant differences across the PFC regions, including optode 1 [F_(2, 268.5)_ = 3.35, *p* < 0.05], optode 3 [F_(2, 310.1)_ = 3.54, *p* < 0.05], optode 5 [F_(2, 311)_ = 4.106, *p* < 0.05], optode 7 [F_(2, 304.5)_ = 5.107, *p* < 0.01], optode 9 [F_(2, 318.8)_ = 4.55, *p* < 0.05], optode 11 [F_(2, 299)_ = 3.26, *p* < 0.05], optode 13 [F_(2, 308.5)_ = 3.52, *p* < 0.05], and optode 16 [F_(2, 317.6)_ = 3.34, *p* < 0.05]. Post hoc analysis showed significant differences between the VR and RW settings in optodes 7 (*p* < 0.01), 3, 5, 9, 11, and 13 (*p* < 0.05) and between the VR and CS settings in optodes 1 and 16 (*p* < 0.05), as depicted in Figure 10. There was also a main effect of the interaction between medium and task difficulty in optode 10 [F_(2, 309.4)_ = 4.95, *p* < 0.01] and optode 11 [F_(2, 297.7)_ = 4.44, *p* < 0.05], which were in the more medial brain area of the right PFC. The results indicate that the participants the solving puzzles in the VR settings showed a higher neural efficiency in terms of PFC activity compared to those solving the puzzles in the other mediums.

## 4. Discussion

In this study, we deployed neuroergonomics techniques to elucidate the neural underpinnings of cognitive effort in visuospatial reasoning. To our knowledge, this is the first study to compare brain activity and behavioral performance in immersive virtual reality, physical reality, and a typical computer-screen-based presentation of the same task. This comparison leveraged the same visuospatial tasks and geometric puzzles performed across differing mediums, offering a new lens through which we evaluated the efficacy of VR technology as an empirically grounded instrument for enhancing spatial abilities. By capturing the nuances of human–computer interaction with these tasks in each environment, we offer substantial evidence for the potential of VR in cognitive training paradigms, paving the way for future educational methodologies.

In the study protocol, we introduced easy and difficult task conditions for each medium. As expected, we observed a significant difference in perceived difficulty between solving the easy and difficult geometry puzzles in the self-assessment survey results, indicating the participants’ ability to differentiate between easy and difficult tasks across the conditions. Such a difference seemed to be clearer in the RW settings in comparison to the VR settings. As expected, fNIRS-based prefrontal brain activity showed a significant difference in the difficulty level in the right medial PFC activity, suggesting that difficult puzzles require more mental effort. This is consistent with a large number of past neuroimaging studies that have indicated that the hemodynamic response in the PFC is related to mental workload in visuospatial task performance [27,32,47,63]. For instance, an fNIRS study showed that subjects in a flight simulation experiment demonstrated heightened hemodynamic activity linked to workload within the anterior PFC area [39], which closely resembled the findings of our neural measures. Another neuroimaging investigation revealed that participants with experience in the Warship Commander Task had relatively lower PFC oxygenation (e.g., less neural activity) at low-to-moderate levels of task load, followed by increased PFC oxygenation during high task loads [64]. Likewise, our fNIRS measures results also indicate that the participants solving the easy puzzles showed a higher neural efficiency in terms of PFC activity compared to those solving the difficult geometric puzzles. These findings show that the level of difficulty we established for each puzzle was in line with the level of complexity assessed by the participants. Furthermore, the data on the brain’s prefrontal region activity corroborate the subjective survey data since the significant difference between hemodynamic activity in the right medial area of the PFC also suggests more cognitive effort in the difficult visuospatial task.

Regarding the medium comparison, the study’s self-assessment survey results suggest a significant difference in the participants’ perceived difficulty between problem-solving in the RW and VR environments. Furthermore, the survey results indicate that the participants reported a higher level of performance and lower mental effort when solving the geometry puzzles in VR compared to in the other mediums. This finding is consistent with our analysis indicating that the employment of VR technology also appears to be a more efficient and prompter medium for participants to solve geometry puzzles than other modalities. The time to solve the puzzles in VR was shorter and the accuracy was higher compared to in the CS and RW environments. A similar study used EEG to measure cognitive load index via alpha and theta oscillations in the frontal and parietal regions, showing that cognitive load was higher when participants engaged in a paper-folding task presented in a 2D projection than in stereoscopic 3D displays [53]. In addition, another study showed that VR-based 3D mental rotation tests resulted in higher scores and faster response times than on-screen 2D stimuli [54], suggesting that perceiving objects in 3D can help alleviate the cognitive challenge of mental rotation tasks. These findings are consistent with our analyses showing that the neural efficiency of the PFC activity was higher during solving the geometry puzzles in the VR environment than in the RW settings.

In fact, VR’s characteristics have been considered helpful in several fields of intervention, from applications in psychology [65,66] to medical treatments [67,68]. Following a similar idea from medical interventions, VR also has the potential to enhance teaching by immersing students in virtual environments. Nicholson et al. [69] showed the educational effectiveness of a 3D anatomical model presented in VR. In this randomized controlled work, students were able to visualize and interact with an inner ear model in contrast with students without access to VR-based technology. Then, both groups answered an anatomy quiz, and their performance was compared. The findings showed that the intervention group’s mean score was significantly better than that of the control group [69]. In addition, many studies have suggested that the hemodynamic activity in the prefrontal cortex region can serve as a reliable indicator during visuospatial tasks, including flight simulators [39,47], spatial navigation [31], tangram puzzles [28,32], and mental rotation tasks [63], which is consistent with our findings on the fNIRS and spatial cognition. VR and fNIRS form a powerful combination that enhances our understanding of spatial cognition.

The analysis of PFC activity for mental effort is a valuable approach to evaluating the efficacy of learning materials, which is particularly useful for comparing various instructional designs [70,71]. Similarly, neural efficiency can also reveal instructional effectiveness in that high-difficulty task performance with low effort indicates high instructional efficiency, while low-difficulty task performance with high effort suggests low instructional efficiency [49]. Notably, the neural efficiency measures in the PFC brain region exhibited regular patterns across the puzzle difficulty levels and all mediums compared in this study. This implies that VR was the medium that relieved the task load, especially for the more cognitively demanding geometry puzzles. One crucial facet of VR lies in its capacity to offer users augmented feedback, referred to as visual cues, through visual, auditory, or even kinesthetic means that supplement the natural feedback [72,73,74]. This added information provided by VR is essential in the process of acquiring knowledge by offering supplementary cues that support the user’s comprehension.

The behavioral data aligned with the information gathered from the subjective survey and fNIRS measurements. For instance, the participants made more actions, rotations, errors, and withdrawals while trying to solve puzzles in the RW, as it was the most challenging medium. The participants also spent more time in the RW attempting to solve the problems, probably leading to a lower neural efficiency. Although interaction is vital to the learning process, excessive attempts to solve problems related to a high difficulty level can exhaust learners and make them give up. This is especially true for beginners. A study using fNIRS showed that novices had higher oxygenation levels at moderate task loads, and such levels dropped significantly at higher task loads, along with performance, indicating disengagement from the task [64]. Our study’s findings suggest that using VR alleviates the visuospatial task load, probably due to facilitating spatial visualization and visual cues. In contrast, the CS as a medium for solving geometry puzzles resulted in lesser interaction between the participants and the task, whereas using the RW led to greater interaction, particularly in the case of more challenging puzzles. It is possible that VR can aid in solving geometrical problems due to its ability to provide visual cues. This, in turn, facilitates the inspection and evaluation of possible solutions for visuospatial problems.

Although the CS setting also employs visual cues to solve the geometry puzzle, the challenge of accurately perceiving the three-dimensional properties of geometric figures, such as depth perception, may hinder one’s performance. In addition to depth perception limitations, viewing 3D pieces on 2D screens might require an extra visuospatial transformation to coordinate the participants’ movements in 2D settings, as demonstrated in other studies [75,76].

Immersive virtual environments have the ability to simulate real-life activities, which can motivate learners [77]. In contrast, desktop virtual environments have limited interaction, as the visual experience is limited to a 2D screen. The higher level of immersion, realism, and interaction in VR environments allows for better adaptation to the sensory needs of learners in real time [78], which is a possible explanation for the better performance in the VR than in CS settings. In fact, using VR-based paradigms provides a higher immersion level than 2D screens, as supported by various studies [79,80,81,82]. Interactions with virtual objects in VR-based settings significantly differ from those in CS settings. For example, the lack of realistic interaction and low immersion could increase cognitive effort during training, negatively impacting learning [76,79]. Present desktop-based 3D stimuli may limit the transferability of newly acquired skills to everyday activities and decrease user motivation to perform 3D tasks on a 2D screen medium.

Data on mental effort and neural efficiency can help in assessing appropriate stimuli in learning and intervention activities. Constructivism-based learning theories have suggested that activities must be challenging to arouse the student’s will to overcome difficulties and find solutions by building new knowledge throughout the learning process [83,84,85]. When the task is too difficult, the student may feel unmotivated. On the other hand, if the task is too easy, the student tends to feel bored [86,87]. An instance of utilizing data from neurotechnology was showcased through our demonstration of how brain activity data obtained from fNIRS can be used to evaluate how visuospatial tasks require cognitive effort in distinct mediums. Regarding brain imaging data in the educational context, measuring cognitive effort could be useful in evaluating interventions, the instructional efficiency of learning materials, and teaching materials to enhance students’ learning experiences [70,71]. By combining biomarkers of workload with behavioral measures, we can make better decisions about how to optimize instructional strategies and improve students’ learning experiences.

VR could be an educational tool to assist students struggling to learn challenging topics, such as those with higher levels of mathematics anxiety who may feel that the anticipation of math is painful [88]. The challenge for instructional designers is that meaningful learning can require heavy cognitive processing, but learners’ cognitive resources are limited [89]. Thus, multimedia instruction should minimize unnecessary cognitive load [89,90]. Considering our findings on VR, it is relevant for teachers and instructors to recognize how the proposed activities can interfere with students’ mental effort during learning and problem-solving. VR offers the opportunity for an interactive learning experience that engages students and helps them understand the concepts they are studying. Educational practice design is essential to ensure that students are not just passively consuming VR content as entertainment. In addition, the technology provides data that can map a student’s performance, enabling teachers to identify areas for improvement and provide personalized instruction guided by objective information.

In light of this discussion, our study suggests that applying the learning activities with the tools of neurosciences could support teaching and learning by applying neurotechnology to unravel students’ mental effort during educational assignments, including geometric domains and visuospatial tasks. Such methods could help evaluate which visuospatial training program would benefit students in terms of developing spatial cognition.

Brain data can be an adjunct in assessing learning and whether an academic activity has an adequate load of difficulty and can even adapt the training with that information for optimal outcomes. For example, a recent study compared learners who received training in flight simulators that were adapted based on their behavioral performance and fNIRS measures combined (called the neuroadaptive group) with those who received traditional training. During the visuospatial task, the neuroadaptive group showed greater efficiency, improved performance, and consistent brain activity patterns of hemodynamic-derived workload in the PFC. The results of this study suggest that personalized neuroadaptive training with fNIRS can enhance learning [39]. In addition to cognitive workload, emotional arousal has been studied with immersive VR with EEG recordings [91,92] and can provide an affective state during VR use for future learning settings. Additionally, VR programs can provide a quantitative measure of session outcomes, individualize training programs, and alter the progression of a training session based on the user’s personal performance [93]. In light of this, VR and fNIRS could be helpful for developing effective interventions. Notably, fNIRS measurements have a lower spatial resolution when compared to fMRI and are limited to a depth of a few centimeters [94]. However, registering to anatomical landmarks and using normalized atlases can improve the inter-participant reliability of fNIRS measures [95]. In addition, many studies have suggested that the hemodynamic activity in the prefrontal cortex region can serve as a reliable indicator during visuospatial tasks, including flight simulators [39,47], spatial navigation [31], tangram puzzles [28,32], and mental rotation tasks [63], which is consistent with our findings on the fNIRS and spatial cognition. VR and fNIRS form a powerful combination that enhances our understanding of spatial cognition.

Our findings suggest that VR applications can facilitate mental imagery by inducing optimal instructions or visual cues for mental imagery or rotations, which may reduce cognitive effort during spatial reasoning. It should be noted that our results do not suggest that performing visuospatial tasks in RW or CS settings is insufficient for developing spatial cognition. This technique is still valid and can be utilized to elevate the difficulty level of the tasks, particularly for experts or individuals who can solve puzzles without visual aids. A potential application of our findings around developing visuospatial skills is that VR would be a helpful tool to assist in spatial cognition training and spatially introducing geometry puzzles to beginners. In addition, it would be possible to progressively increase the task’s challenge level by using RW puzzles as training progresses after the person demonstrates mastery in VR problem-solving. This approach would be a method to maintain a level of progressive challenge throughout spatial cognition training. Assessing brain activity in real-world scenarios holds great significance in the field of neuroergonomics [27,45]. Neuroergonomic approaches provide perspectives for enhancing the efficacy and configuration of interrelations between humans and systems focusing on everyday life situations [96,97,98]. Investigations into assessing cognitive workload serve as a driving force behind research on neuroergonomic professional training in critical settings [27], such as surgeons operating in a VR environment (see [99,100] for review), pilots, and air traffic controllers [39,101]. Similarly, we applied the neuroergonomics method to provide insights into the relationship between brain function and behavioral outcomes. Developing systems that adapt user interfaces or interventions based on the continuous display of the users’ mental states via neurofeedback techniques could be facilitated through further investigations [26,29]. Also, it is possible to measure the brain data of patients using VR as a rehabilitation tool [102,103,104,105].

As a limitation, this study included only one session. It would be interesting to conduct an experiment investigating the training effect on mental effort and performance. In order to fully understand the potential benefits of incorporating VR and spatial cognition into education, conducting a study with a group of students is crucial. Through a longitudinal study, we could gain insight into the adaptation over time. Furthermore, the study participants included a small sample of healthy adults. In order to enhance the conclusiveness and generalizability of the findings, further investigations should incorporate a larger sample size and extend the exploration to a broader population. Future studies could apply the methodologies described here to investigate the impact of the medium in pediatric populations. It is also important to monitor the development of the student’s ability to solve geometric problems during their training sessions, both with and without VR. This would allow us to better understand any changes in the cortical regions activated during visuospatial tests. Furthermore, we can evaluate the effectiveness of the VR training program across other domains, such as MRT and mathematical problem-solving, and could potentially offer this training to students who may be struggling with STEM subjects to assess its impact on academic performance. Finally, the main goal of this study was to compare brain activity and behavioral performance in immersive virtual reality, physical reality, and a typical computer screen-based presentation of the same task. Further studies should investigate the relationship between gender, age, or game familiarity with fNIRS data, performance, or a self-report questionnaire. These are all vital questions that warrant further exploration in future research.

## 5. Conclusions

To our knowledge, this neuroergonomic study is the first of its kind to compare subjective effort, neural activity, and performance in visuospatial tasks across three different mediums: VR, computer screen, and the physical real world. The insights derived from this research shed light on the impact of different task visualization technologies on cognitive load and technology usability, potentially influencing the development of visuospatial skills. Our findings not only contribute to the understanding of the effects of different mediums but also unveil the promising potential of fNIRS in advancing methodologies within VR applications and science-based tools for visuospatial skill development. The implications of our research suggest the opportunity to enhance visuospatial training programs through immersive virtual environments. This study reveals that VR is not only a more effective platform for engaging with geometry puzzles but also enhances precision compared to traditional computer screens and real-world presentations. The innovative use of fNIRS to monitor brain activity indicates that the prefrontal cortex operates with greater neural efficiency during VR-mediated visuospatial tasks, emphasizing VR’s ability to streamline cognitive processing and enhance mental imagery. These observations collectively suggest the adoption of VR technologies as a useful tool in the realm of cognitive training, particularly for tasks requiring spatial reasoning.

## Data Availability

The original contributions presented in the study are included in the article/supplementary material, further inquiries can be directed to the corresponding author/s.
